# Exome-based investigation of the genetic basis of human pigmentary glaucoma

**DOI:** 10.1186/s12864-021-07782-0

**Published:** 2021-06-26

**Authors:** Carly van der Heide, Wes Goar, Kacie J. Meyer, Wallace L. M. Alward, Erin A. Boese, Nathan C. Sears, Ben R. Roos, Young H. Kwon, Adam P. DeLuca, Owen M. Siggs, Claudia Gonzaga-Jauregui, Val C. Sheffield, Kai Wang, Edwin M. Stone, Robert F. Mullins, Michael G. Anderson, Bao Jian Fan, Robert Ritch, Jamie E. Craig, Janey L. Wiggs, Todd E. Scheetz, John H. Fingert

**Affiliations:** 1 Department of Ophthalmology and Visual Sciences, Carver College of Medicine, 3111B Medical Education and Research Facility, University of Iowa, 375 Newton Road, Iowa City, IA52245 USA; 2 Institute for Vision Research, University of Iowa, Iowa City, IA USA; 3 Department of Molecular Physiology and Biophysics, Carver College of Medicine, University of Iowa, Iowa City, IA USA; 4 Department of Ophthalmology, Flinders Medical Centre, Adelaide, South Australia Australia; 5 Garvan Institute of Medical Research, Darlinghurst, NSW Australia; 6 Regeneron Genetics Center, Regeneron Pharmaceuticals Inc., Tarrytown, NY USA; 7 Department of Pediatrics, Carver College of Medicine, University of Iowa, Iowa City, IA USA; 8 Department of Biostatistics, College of Public Health, University of Iowa, Iowa City, Iowa USA; 9 VA Center for the Prevention and Treatment of Visual Loss, Iowa City VA Healthcare System, Iowa City, IA USA; 10 Department of Ophthalmology, Harvard Medical School, Massachusetts Eye and Ear Boston, Boston, MA USA; 11 Einhorn Research Center, New York Eye and Ear Infirmary of Mount Sinai, New York, NY USA

**Keywords:** Pigmentary Glaucoma, Pigment dispersion syndrome, Glaucoma, Exomes, Human genetics

## Abstract

**Background:**

Glaucoma is a leading cause of visual disability and blindness. Release of iris pigment within the eye, pigment dispersion syndrome (PDS), can lead to one type of glaucoma known as pigmentary glaucoma. PDS has a genetic component, however, the genes involved with this condition are largely unknown. We sought to discover genes that cause PDS by testing cohorts of patients and controls for mutations using a tiered analysis of exome data.

**Results:**

Our primary analysis evaluated melanosome-related genes that cause dispersion of iris pigment in mice (*TYRP1*, *GPNMB*, *LYST*, *DCT*, and *MITF*). We identified rare mutations, but they were not statistically enriched in PDS patients. Our secondary analyses examined *PMEL* (previously linked with PDS), *MRAP*, and 19 other genes. Four *MRAP* mutations were identified in PDS cases but not in controls (*p* = 0.016). Immunohistochemical analysis of human donor eyes revealed abundant MRAP protein in the iris, the source of pigment in PDS. However, analysis of *MRAP* in additional cohorts (415 cases and 1645 controls) did not support an association with PDS. We also did not confirm a link between *PMEL* and PDS in our cohorts due to lack of reported mutations and similar frequency of the variants in PDS patients as in control subjects.

**Conclusions:**

We did not detect a statistical enrichment of mutations in melanosome-related genes in human PDS patients and we found conflicting data about the likely pathogenicity of *MRAP* mutations. PDS may have a complex genetic basis that is not easily unraveled with exome analyses.

**Supplementary Information:**

The online version contains supplementary material available at 10.1186/s12864-021-07782-0.

## Background

Pigment dispersion syndrome (PDS) is a common eye condition that is characterized by an abnormal release of iris pigment in as many as 2.5% of the general population in the United States [1]. PDS is most common among young (20 to 40-year-old), myopic Caucasians and is associated with a concave iris configuration, which promotes pigment shedding due to mechanical rubbing of the iris pigmented epithelium against posterior structures (Campbell, 1979). Liberated iris pigment accumulates within the eye including within the tissues where fluid drains from the eye, the trabecular meshwork. Pigment deposition is hypothesized to increase intraocular pressure (IOP) by damaging trabecular meshwork endothelial cells and decreasing aqueous humor outflow [2, 3]. The risk for patients with PDS to develop elevated IOP and secondary glaucoma, called pigmentary glaucoma (PG), is estimated to be 10% at 5 years, and 15% at 15 years [4], although rates as high as 50% have been reported [5–8].

PG has a strong genetic basis. Characteristic clinical features of PDS are inherited in a Mendelian fashion among inbred dogs [9], mice [10, 11], and in rare human pedigrees with autosomal dominant inheritance [12–15], suggesting that genetics are likely a component of PDS etiology. Family-based studies using positional cloning approaches have identified loci linked to autosomal dominant familial cases of PDS [13, 16], however, the causative genes in these loci have not yet been identified. Most recently, a whole exome analysis of pedigrees with PDS identified mutations in the premelanosome protein (*PMEL*) gene that were detected in 2.1 to 3.5% of PDS and PG cases [12].

Studies of several inbred strains of mice that develop iris pigment release similar to human PDS have suggested that genes involved in melanin synthesis are potential candidates for causing human disease. Mutations in *Tyrp1* and *Gpnmb* cause progressive release of pigment from the posterior iris (iris pigment epithelium) in DBA/2 J mice [11, 17, 18]. C57BL/6 J mice with the *beige* mutation have marked pigment dispersion and highly enlarged melanosomes due to a mutation in *Lyst* (lysomal trafficking regulator gene) [19–21]. Two other mutations, *nm2798* and *vitiligo*, lead to pigment dispersion as a result of mutations in two other genes involved in melanin synthesis, *Dct* and *Mitf*, respectively [19]. Mutations influencing these melanosomal proteins may impair melanogenesis and lead to a buildup of highly cytotoxic intermediates [22], ultimately leading to cell death in the iris and release of pigment into aqueous humor.

We hypothesized that mutations in genes associated with pigment dispersion in animals (*TYRP1, GPNMB, LYST, DCT* and *MITF*) may be associated with PDS in humans. To test our hypothesis, we screened a cohort of 201 patients with PDS and 360 control subjects for mutations in these 5 candidate genes as a primary outcome of a whole exome sequencing study. We also performed secondary analyses of the variants we detected in other genes.

## Results

### Cohort of PDS patients (cohort 1, Iowa)

A cohort of 210 patients were diagnosed with PDS based on the presence of classic features of disease: 1) iris transillumination defects; 2) dispersed iris pigment on the cornea (Krukenberg spindle); 3) dispersed iris pigment on the lens zonules (Scheie Stripe or Zentmayer ring); and 4) dispersed iris pigment in the trabecular meshwork. Patients with 2 or more of these features were included in the PDS cohort [23]. A total of 148 (70%) of 210 PDS patients had optic nerve cupping and corresponding glaucomatous visual field defects and were additionally diagnosed with PG. The demographic and clinical features of these patients are shown in Table 1.

**Table 1:** Clinical features of PDS and control patient cohorts from Iowa

	PDS cohort	Control cohort
	***n*** = 210	***n*** = 362
**Age**^a^	59.6 ± 13.5	61.1 ± 20.2
**Gender (female %)**	33.8%	56.4%
**Self-reported race / ethnicity**^**2**^		
White (European ancestry)	97.55%	97.60%
Black (African ancestry)^3^	0.98%	0.60%
Hispanic	0.49%	0%
Asian (Chinese)	0.49%	0.60%
Native American / Alaskan	0.49%	1.2%
**Signs of PDS detected**		
Krukenberg spindle	175 (83%)	
Heavy pigmentation of trabecular meshwork	198 (94%)	
Iris transillumination defects	180 (86%)	
Scheie stripe	35 (17%)	
**Diagnosis**		
PDS suspect	5 (2.4%)	
PDS	26 (12.4%)	
PDS with OHT	21 (10%)	
Pigmentary glaucoma with OHT	148 (70%)	
Pigmentary glaucoma without OHT	3 (1.4%)	
Other (excluded from analysis)	7 (3.3%)	
**Genetics study participants**		
Total enrolled	210	362
Excluded for incomplete clinical records	7	0
Excluded for relatedness to another subject	1	1
Excluded for a secondary cause of pigment release	2	0
Excluded as outliers based on PCA	2	2
Total investigated with genetic studies	198	359

^a^Age is at the time of blood draw and was available for *n* = 190 PDS patients (90%) and *n* = 348 control subjects (96%). ^2^Self-reported race/ethnicity was available from 97% of cases and 46% of controls. ^3^Subsequently eliminated from the study as outliers on principal components analysis. Abbreviations in the table are: ocular hypertension (OHT)

### Evaluation of five candidate genes for loss-of-function mutations in PDS cases and controls (primary analysis)

We studied 198 PDS patients and 359 controls with complete clinical records and ethnicity matched by principal components using whole exome analysis. We first investigated loss-of-function mutations, i.e. nonsense, frameshift, and canonical-splice variants, in five genes that have been previously associated with pigment dispersion in mice, *TYRP1* [10], *GPNMB* [10], *LYST* [21], *DCT* [19], and *MITF* [19]. We compared the frequency of loss-of-function mutations in these candidate genes between our PDS patients and controls using Fisher’s exact test. A single instance of a loss-of-function mutation was detected in each of the *TYRP1*, *GPNMB*, and *LYST* genes and no mutations were detected in *DCT* and *MITF*. The mutations in *TYRP1* and *LYST* were all detected in the PDS cohort, while the *GPNMB* mutation was detected in the control cohort. Statistical analysis of each of these mutations demonstrated no association with PDS in our cohort (*p* > 0.01). Each of these mutations is described in more detail in Table 2.

**Table 2:** Loss-of-function mutations in candidate genes

				PDS Cohort		Normal Controls			gnomAD v2.1.1
		**Loss of function mutation**		**n = 198**		**n = 359**			**European (non-Finnish)**
**Gene**	**SNP ID**	**Mutation**	**Encoded protein**	**Instances**	**Genotype frequency**	**Instances**	**Genotype frequency**	**P-value**	**Genotype frequency**
*LYST*	NA	NM_001301365.1:c.8501C > A	p.Ser2834Ter	1	0.51%	0	0	0.36	0%
*TYRP1*	rs749735228	NM_00550.3:c.410_413dupGTAA	p.Glu139Ter	1	0.51%	0	0	0.36	0.0017%
*GPNMB*	rs758729806	NM_002510.3: c.310_323delAGATGCCAAAAGGA	p.Lys107TrpfsTer6	0	0	1	0.28%	> 0.99	0.16%

### Secondary analysis #1. Evaluation of 21 potential candidate genes for loss-of-function mutations in PDS cases and controls

A panel of 21 additional genes (Supplemental Table 1) that are potential candidates for causing PDS (*AGRP*, *ATP6V1A*, *ATP6V1B2*, *COMT*, *CYP5R3*, *EN2*, *GPR143*, *LAMP1*, *LAMP2*, *MC1R*, *MIF*, *MLANA*, *MNX1*, *PAXIP1*, *PMEL*, *POMC*, *RACK1*, *SHH*, *SLC45A2*, *TYR*, and *VAT1*) was assembled based on their function in melanin synthesis, melanosome biology, or their location within the previously identified PDS locus GPDS1 [13].

We next analyzed whole exome sequence data from our cohort of 198 PDS patients and 359 control subjects for mutations in these 21 potential candidate genes using the same filtration strategy to find loss-of-function mutations as was used in the primary analysis (Supplementary Table 2). We detected heterozygous variants in three genes: melanocortin 1 receptor (*MC1R*), solute carrier family 45 member 2 (*SLC45A2*), and tyrosinase (*TYR*). A single instance of a frameshift variant in the *MC1R* gene (p.Ile180HisfsTer59) and a single instance of a nonsense variant in the *TYR* gene (p.Arg116Ter) were each detected in PDS patients. One variant, a frameshift mutation in *SLC45A2* (p.Gly89AspfsTer24), was identified in a normal control subject. These rare variants were present in a European non-Finnish population in the gnomAD database (*MC1R* p.Ile180HisfsTer59 with a genotype frequency of 0.10%; *SLC45A2* p.Gly89AspfsTer24 with a genotype frequency of 0.01%; and *TYR* Arg116Ter with a genotype frequency 0.001%) and were not associated with PDS in our cohort (uncorrected *p* > 0.05).

Mutations in human PDS patients were recently reported in one of these 21 genes, *PMEL* [12] after we had designed and begun our study. We detected no loss-of-function mutations in *PMEL* as part of our planned analysis. A total of 7 instances of 5 unique *PMEL* mutations were detected (Table 3) that were not statistically more common in PDS patients than in controls (*p* > 0.99). Moreover, those *PMEL* variants predicted to be damaging to protein function were found exclusively in the control patients in our study.

**Table 3:** PMEL mutations

						PDS Cohort		Normal Controls		gnomAD
			**Mutation analysis**			**n = 198**		**n = 359**		**European (non-Finnish)**
**SNP ID**	**PMEL Mutation**		**SIFT**	**PolyPhen**	**Blosum62**	**Instances**	**Genotype frequency**	**Instances**	**Genotype frequency**	**Genotype frequency**
rs750040742	NM_001320121.1:c.515 T > C	p.Ile172Thr	Deleterious	Possibly Damaging	-1	0	0%	1	0.28%	0.015%
rs200641128	NM_001320121.1:c.574C > T	p.Arg192Trp	Deleterious	Possibly Damaging	−3	0	0%	1	0.28%	0.043%
NA	NM_001320121.1:c.668G > A	p.Arg223Gln	Tolerated	Benign	1	1	0.51%	0	0%	0.025%
rs148568175	NM_001320121.1:c.686A > G	p.Asn229Ser	Tolerated	Benign	1	1	0.51%	2	0.56%	0.47%
NA	NM_001320121.1:c.812C > A	p.Ser271Tyr	Deleterious	Probably damaging	−2	0	0%	1	0.28%	0%
**Total**						**2**	**1.0%**	**5**	**1.4%**	
						**p > 0.99**				

### Secondary analysis #2: genome-wide scan for rare non-silent mutations using mutation burden analysis (SKAT-O)

We further identified all non-silent mutations, i.e. missense, nonsense, frameshift, canonical spice site, and in-frame deletion/insertion mutations in our cohorts of PDS patients and control subjects in each of 17,253 genes of the exome that met quality control requirements. In this analysis, we compared the non-silent mutation burden between the PDS cohort and the control cohort using SKAT-O for each gene. Uncorrected *P*-values were calculated to prioritize candidate genes that may have a role in PDS pathophysiology (Supplemental Table 3).

The melanocortin 2 receptor accessory protein (*MRAP*) gene was the top candidate from this analysis. We identified four instances of three unique non-silent *MRAP* mutations in PDS patients and no non-silent mutations in 359 control subjects, *p* = 0.016 (Table 4). One mutation NM_206898.1:c.302C > T (rs140113354) that causes a conservative amino acid substitution p.Ala101Val was detected in three (1.5%) of our cohort of 198 PDS patients and was absent from our controls but present in 3.7% of South Asians in the gnomAD v2.1.1 database (https://gnomad.broadinstitute.org/) and was therefore excluded from our analysis. The p.Ala101Val mutation had a neutral BLOSUM62 score of 0 and was judged *possibly damaging* by Polyphen and *tolerated (low confidence)* by SIFT. Another mutation, NM_178817.4:c.18C > A (rs138040820), that produces a less conservative amino acid substitution p.Asn6Lys was identified in 2 (1.0%) of 198 PDS patients and in none of our control subjects. However, this mutation was detected in the gnomAD v2.1.1 database at 0.10% in Non-Finnish Europeans. The p.Asn6Lys has a BLOSUM62 score of 0 and was judged *probably damaging* by Polyphen and *deleterious* by SIFT. Another notable variant was the *MRAP* transcript, NM_178817.4:c.3G > A (rs80358231) that eliminates the start codon p.Met1? and might alter gene expression dramatically. One (0.51%) of 198 PDS patients carried the p.Met1? mutation, which was not identified in our normal control subjects but was present in 0.019% of Non-Finnish Caucasians in the gnomAD database. Each of these rare *MRAP* variants was detected at a higher frequency in our PDS patient cohort than in our control cohort or than in the gnomAD database. However, it is also notable that some *MRAP* mutations detected in the Iowa cohort were observed at dramatically higher frequencies in populations of African or Asian ancestry compared to Non-Finnish European populations in the gnomAD v2.1.1 database (Table 4).

**Table 4:** MRAP mutations detected in PDS cases

						Cohort 1				Cohort 2										Cohort 1 and 2				gnomAD v2.1.1				
						IA PDS		IA Normals		NY EEI PDS		MEE PDS		MEE Controls		Australian PDS		Australian Controls		Total Cases		Total Controls		Non-Finnish European	African and African American	South Asian		
**Mutations detected**						**n = 198**		**n = 359**		**n = 88**		**n = 150**		**N = 1500**		**n = 177**		**n = 145**		***n*** **= 613**		***N*** **= 2004**		***n*** **> 55,000**	***n*** **> 24,000**	***N*** **> 15,000**		
**SNP ID**	**MRAP Mutation**		**SIFT**	**PolyPhen**	**Blosum62**	**Instances**	**Genotype frequency**	**Instances**	**Genotype frequency**	**Instances**	**Genotype frequency**	**Instances**	**Genotype frequency**	**Instances**	**Genotype frequency**	**Instances**	**Genotype frequency**	**Instances**	**Genotype frequency**	**Instances**	**Genotype frequency**	**Instances**	**Genotype frequency**	**Genotype frequency**	**Genotype frequency**	**Genotype frequency**		
rs80358231	NM_178817.4:c.3G > A	p.Met1?	Deleterious	Possibly damaging	NA	1	0.51%	0	0%	0	0%	0	0%	0	0%	0	0%	0	0%	1	0.16%	0	0%	0.019%	0.016%	0%		
rs138040820	NM_178817.4:c.18C > A	p.Asn6Lys	Deleterious	Probably damaging	0	2	1.0%	0	0%	0	0%	0	0%	2	0.13%	0	0%	0	0%	2	0.33%	2	0.10%	0.10%	0.040%	0%		
rs781703497	NM_178817.4:c.190G > A	p.Ala64Thr	Deleterious	Benign	0	0	0%	0	0%	0	0%	1	0.67%	0	0%	0	0%	0	0%	1	0.16%	0	0%	0.0031%	0.0080%	0.059%		
rs200277269	NM_178817.4:c.247G > A	pGly83Ser	Tolerated	Benign	−1	0	0%	0	0%	0	0%	0	0%	1	0.067%	0	0%	0	0%	0	0%	1	0.050%	0.0078%	0.21%	0.0065%		
NA	NM_206898.1:c.301G > A	p.Ala101Thr	Tolerated	Benign	0	1	0.51%	0	0%	0	0%	0	0%	0	0%	0	0%	0	0%	1	0.16%	0	0%	0%	0%	0%		
rs139379303	NM_178817.4:c.322G > A	p.Ala108Thr	Tolerated	Benign	0	0	0%	0	0%	0	0%	0	0%	3	0.20%	0	0%	0	0%	0	0%	3	0.15%	0.011%	0%	0%		
rs200921993	NM_178817.4:c.359A > C	p.Asp120Ala	Tolerated	Benign	−2	0	0%	0	0%	0	0%	0	0%	12	0.80%	0	0%	0	0%	0	0%	12	0.60%	0.78%	1.1%	0.019%		
rs200448756	NM_178817.4:c.446A > G	p.Asn149Ser	Tolerated	Benign	1	0	0%	0	0%	0	0%	0	0%	0	0%	0	0%	1	0.69%	0	0%	1	0.050%	0.0079%	0.0081%	0%		
rs142897309	NM_178817.4:c.508 T > A	p.Leu170Met	Deleterious	Possibly damaging	2	0	0%	0	0%	0	0%	0	0%	2	0.13%	0	0%	0	0%	0	0%	2	0.10%	0.011%	0.57%	0%		
**Total**						**4**	**2.0%**	**0**	**0%**	**0**	**3.4%**	**1**	**0.67%**	**20**	**1.3%**	**0**	**0%**	**1**	**0.69%**	**5**	**0.82%**	**21**	**1.0%**					
**Fisher’s Exact Test**						**p = 0.016**				**NA**		**p = 0.71**				***p*** **= 0.45**				**NA**								
**Cochran-Mantel-Haenszel**										**p = 0.49**										**p = 0.71**								
**Mutations detected but excluded due to prevalence in control populations (> 2.5%)**																												
rs75858661	NM_178817.4:c.148G > A	p.Val50Met	Deleterious	Probably damaging	1	0	0%	0	0%	3	3.4%	1	0.67%	3	0.20%	1	0.57%	0	0%	5	0.82%	3	0.15%	0.029%	**8.0%**	0.020%		
rs79126334	NM_178817.4:c.234C > G	p.Cys78Trp	Deleterious	Probably damaging	−2	0	0%	0	0%	0	0%	2	1.3%	6	0.40%	0	0%	0	0%	2	0.33%	6	0.30%	0.017%	**11%**	0.0065%		
rs115917390	NM_206898.1:c.257G > T	p.Arg86Leu	Tolerated	Benign	−2	0	0%	0	0%	0	0%	1	0.67%	4	0.27%	0	0%	0	0%	1	0.16%	4	0.20%	0.017%	**5.6%**	0.0065%		
rs140113354	NM_206898.1:c.302C > T	p.Ala101Val	Tolerated	Benign	0	3	1.5%	0	0%	0	0%	0	0%	14	0.93%	0	0%	0	0%	3	0.49%	14	0.70%	0.51%	0.04%	**3.7%**		
rs114530014	NM_178817.4:c.389C > T	p.Thr130Ile	Deleterious	Benign	−1	0	0%	0	0%	0	0%	0	0%	10	0.67%	1	0.57%	0	0%	1	0.16%	10	0.50%	0.022%	**9.8%**	0%		

These variants in the bottom section were detected at a frequency > 2.5% in control populations of Non-Finnish European, African, or South Asian ancestry (gnomAD database) and were excluded from further analysis. Abbreviations in the table are: Iowa (IA), Massachusetts Eye and Ear (MEE), New York Eye and Ear Infirmary (NYEEI)

### Analysis of a second cohort of PDS patients and controls for *MRAP* mutations

A second cohort was comprised of 150 PDS patients and 1500 control subjects from Massachusetts Eye and Ear (MEE); 88 PDS patients from the New York Eye and Ear Infirmary (NYEEI); and 177 PDS patients and 145 control subjects from the Flinders Medical Centre, South Australia. We tested this cohort for *MRAP* mutations and detected a total of 75 instances of 14 unique variants (Table 4). The majority of the *MRAP* variants that were identified in PDS patients were also observed in the control cohorts, though at lower frequency and most detected variants were excluded due to their high prevalence in the gnomAD databases. The frequency of non-synonymous mutations in *MRAP* was the same in PDS cases and controls (*p* = 0.49, Mantel-Haenszel test). Moreover, when data from all cohorts are combined, the frequency of *MRAP* mutations is not statistically different between PDS cases (0.82%) and controls (1.0%) (*p* = 0.71, Mantel-Haenszel test).

### Investigation of *MRAP* as a PDS candidate gene (Immunohistochemical analysis in human iris

**Fig. 1:**
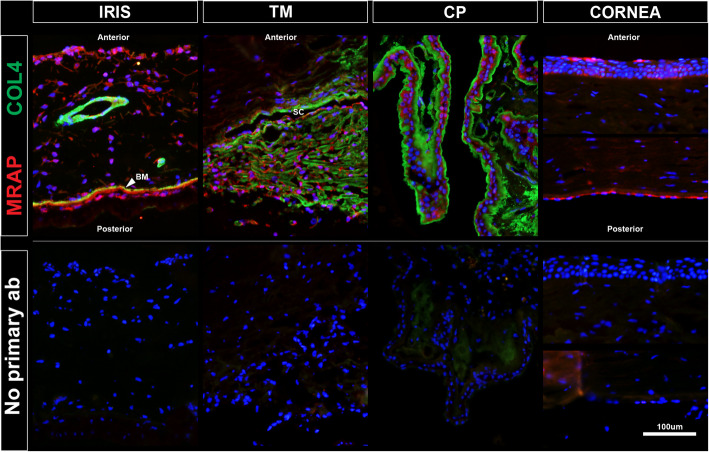
MRAP immunoreactivity in the human eye. A section of anterior segment from a human eye was immunolabeled (top row) with anti-MRAP antibody (red) and anti-collagen IV antibody (green). Nuclei were stained with DAPI (blue). MRAP immunoreactivity was observed throughout anterior segment structures, including the iris, trabecular meshwork (TM), ciliary processes (CP) and cornea. Immunopositive cells were observed on the anterior surface of the iris, throughout the iris stroma, and on the posterior aspect of the basement membrane (BM). Strong immunoreactivity was also exhibited by trabecular meshwork cells, including the endothelial lining of Schlemm’s canal (SC). Epithelial cells of ciliary processes and corneal epithelium and endothelium were also immunopositive. A section from the same eye was treated identically but without the addition of primary antibodies to serve as a control for non-specific labeling by secondary antibodies (bottom row)

To further explore *MRAP* as a candidate gene for PDS, we determined the ocular expression of MRAP using immunohistochemical analysis of human donor eyes. Strong MRAP immunoreactivity was observed in the iris, trabecular meshwork, ciliary processes, and cornea (Fig. 1). Within the iris, immunopositive cells were observed along the anterior surface, dispersed throughout the stroma, and along the posterior surface of the basement membrane. Of the two layers of posterior iris pigmented epithelia, the anterior layer (in close contact with the basement membrane) had much stronger immunoreactivity, while the posterior layer was weakly immunopositive. In the trabecular meshwork, most cells were immunopositive, including the endothelial lining of Schlemm’s canal. In the ciliary body, the ciliary epithelial cells were strongly immunopositive. Within the cornea, the epithelial layer on the anterior surface as well as the endothelial layer on the inner surface were both strongly immunoreactive.

## Discussion

The genetic basis of PDS and PG is largely unknown. Several large pedigrees that exhibit autosomal dominant inheritance of PDS have been reported [12, 13, 23–25], suggesting that some cases of disease are caused by mutations in single genes. Most recently, studies of two moderately-sized pedigrees led to the discovery of the *PMEL* mutations in 2% of PDS patients [12]. Many more likely remain to be discovered.

We sought to identify more PDS genes with a tiered analysis of whole exome data. The primary analysis of our study was to search for disease-causing mutations in five candidate genes *TYRP1* [10], *GPNMB* [10], *LYST* [21], *DCT* [19], and *MITF* [19]. Several rare mutations were detected in our PDS cohort, however, these mutations were not statistically enriched in our PDS cohort. Although our study was not able to demonstrate a statistically significant result, the rare variants discovered may still have value. It is possible that further study of the rare *TYRP1* and *LYST* mutations we detected might provide new insights into the basic biology of melanosomes, pigmentation and the pathogenesis of PDS. Future studies of these rare variants have the potential to provide more general insights about TYRP1 and LYST protein function and biological pathways that are important in pigmentation, PDS, and the development of glaucoma.

Secondary analyses of the whole exome data collected from our PDS patients and control subjects led to several interesting discoveries. We evaluated our cohort for mutations in the *PMEL* gene. Although we did detect some rare variants in *PMEL* (Table 3), they occurred at the same frequency in PDS patients as in control subjects. Moreover, those *PMEL* mutations with likely pathogenic SIFT, PolyPhen, and BLOSUM62 scores were only detected in our control population (Table 3). We did not detect any of *PMEL* mutations that were previously reported in PDS patients [12]. Unfortunately, our analysis of *PMEL* in the cohorts from Iowa does not provide confirmatory evidence for its role in PDS. It is possible that we were unable to replicate the association between mutations in *PMEL* and PDS due to differences in the composition of the patient cohorts. *PMEL* mutations might be less prevalent in our cohort from Iowa due to differences in the ethnic and geographic origin. Alternatively, the patients in our study may have had a weaker family history of PDS that may have biased our study against finding a genetic association. The role of *PMEL* mutations in PDS pathogenesis will likely be clarified as more cohorts are analyzed in future studies.

We detected several rare mutations in *MRAP* that were enriched in our cohort of PDS patients from Iowa and absent from matched control subjects (Table 4), which suggests that they may contribute to PDS. Moreover, we demonstrated MRAP protein is most abundant in the highly pigmented layer of the iris, the iris pigment epithelium, which is also the site of pigment release in PDS (Fig. 1). These data provide compelling evidence that *MRAP* mutations might cause some cases of PDS. We attempted to confirm our mutation screening data by testing a second cohort of PDS patients and controls from MEE, NYEEI, and Australia for *MRAP* mutations. Although we did detect *MRAP* mutations in the PDS patients from additional cohorts, they were also present in control subjects at the same frequency. Future studies with more patients and/or investigations that employ functional assays might be able to demonstrate that some *MRAP* mutations cause PDS, however, currently there is conflicting data about their likely pathogenicity.

This study has limitations. Although our study is the largest whole exome study of PDS and PG completed to date, the sample size of our study is still relatively small and may have limited the ability to detect rare disease-causing variants. Although all control subjects were examined by board-certified ophthalmologists to rule out glaucoma and other ophthalmic disease, it is possible that some had subtle findings of PDS that were either not recognized at the time of examination or that developed some time after enrollment. Such features of our control population may have also reduced the power of our study to find genes that cause PDS. Many of the detected mutations have been observed at dramatically different frequencies in different ethnic populations, which indicates the potential for stratification bias. Even though we had well-matched case and control cohorts as evidenced by principal components analysis, stratification bias remains a possibility. Another potential confounder is incomplete penetrance. It is possible that incomplete penetrance of mutations in *MRAP* (or *PMEL*) may hindered attempts to detect an association with PDS. We focused our primary analysis on loss-of-function mutations in an attempt to better identify variants likely to be pathogenic, it is possible that a less stringent criteria for mutations that included missense mutations may have been more successful. Finally, it is possible that some cases of PDS are familial and caused by inheritance of a mutation in a single gene, while other cases are caused by more complex interaction of many genetic and environmental factors. Our exome analysis searched for rare variants in single genes as potential causes of PDS but was not designed or equipped to identify polygenic causes of PDS.

## Conclusions

We report an exome-based analysis of PDS patients and controls. Our primary analysis of loss-of-function mutations in five genes related to pigmentation (*TYRP1*, *GPNMB*, *LYST*, *DCT*, and *MITF*) did not identify a significant enrichment of loss-of-function mutations in PDS patients. We further evaluated the *PMEL* gene for disease-causing mutations and were unable to confirm the previous report linking this gene with PDS with our analysis. Prior reports indicated that non-synonymous *PMEL* mutations alter processing of PMEL protein in cell culture [12] and this is an area of potential future study of PDS pathogenesis. Finally, we discovered missense mutations of the *MRAP* gene that are enriched in one of two cohorts of PDS patients. We also demonstrated that MRAP protein is abundantly produced within the tissues of the human eye that are most affected by PDS including the iris pigment epithelium. Together these data suggest that *MRAP* may have a role in the pathogenesis of PDS, however, analysis of *MRAP* in a second cohort was not confirmatory of an association with PDS. Although the prevalence studies are contradictory, functional and/or animal studies may be able to determine if some of the rare *MRAP* mutations detected are in fact pathogenic. The small number of PDS-causing genes that have been discovered to date suggest that most cases of PDS have a complex genetic basis not easily recognized by exome-based analyses without much larger cohorts.

## Methods

### Patient enrollment

The primary analysis was conducted with a case-control cohort from Iowa and confirmatory studies were conducted using cohorts from Massachusetts, New York, and Australia described below.

#### Iowa cohort

This study was approved by the Institutional Review Board at the University of Iowa and adhered to the tenets of the Declaration of Helsinki and the ARVO statement on human subjects. All study subjects provided written informed consent and were enrolled at the University of Iowa Department of Ophthalmology and Visual Sciences. Study subjects were evaluated for PDS with a complete eye examination including slit lamp examination, gonioscopy, and ophthalmoscopy. In some cases, infra-red iris transillumination was performed to search for subtle signs of iris transillumination defects. Patients were diagnosed with PDS if they had two of four principal features of this condition: radial iris transillumination defects, Krukenberg spindle, Scheie stripe, and moderate to heavy pigmentation of the trabecular meshwork (≥2+ pigmentation on gonioscopy) as we have previously described [23]. Control subjects were enrolled from the same ophthalmology clinics at the University of Iowa and were examined by a board-certified ophthalmologist and judged not to have glaucoma or ocular hypertension. Although control subjects were not specifically examined for the presence of PDS and they were not examined with infra-red iris transillumination, they were also not noted to have classic signs of disease (Krukenberg’s spindle, iris transillumination defects, or heavily pigmented trabecular meshwork) [26].

A total of 210 cases of PDS and 362 controls were enrolled. Self-reported race/ethnicity was available from 204 (97%) of 210 cases and from 167 (46%) of controls (Table 1). However, 9 of the 210 PDS patients in the Iowa cohort were excluded from analysis due to incomplete clinical records (7 subjects), presence of iris cysts (1 subject), or presence of a sulcus intraocular lens (1 subject). Thus, 201 PDS patients and 362 controls were available for genetic study. DNA was obtained from blood samples using standard techniques as we have previously described [27].

#### New York eye and ear Infirmary (NYEEI) cohort

A second cohort of patients with PDS (*n* = 88) from NYEEI was used to replicate results from analysis of Iowa patients and controls. Patients were judged to have PDS based on identification of Krukenberg’s spindle, iris transillumination defects, and heavily pigmented trabecular meshwork on clinical exam.

#### Massachusetts eye and ear (MEE) cohort

A third cohort of patients with PDS (*n* = 150) and control subjects from MEE (*n* = 1500) was also used to replicate our analysis of Iowa patients and controls. The diagnostic criteria for the MEE cohorts and their clinical features have been previously described [12].

#### Australian cohort

A fourth cohort of patients with PDS (*n* = 177) and control subjects (*n* = 145) from the Flinders Medical Center in Adelaide, Australia was also used for replication studies. Enrollment criteria for this cohort has also been previously described [12].

### DNA sequencing

Whole exome sequencing of the Iowa cohort was performed on PDS patient and control subject DNA at the same time using the same capture system as previously described [28] in collaboration with Regeneron, Inc. DNA was fragmented using sonication. Library preparation and sample bar-coding was accomplished with KAPA reagents (KAPA Biosystems) and exome capture was conducted using SeqCap VCRome probes (Nimblegen). Paired-end sequencing was performed on an Illumina HiSeq 2500. DNA sequence reads were aligned to the human reference genome GRCh37 using the Burrows-Wheeler aligner [29]. Sequence variants were identified using the Genome Analysis Tool Kit (GATK) [30] and a custom sequence analysis and annotation pipeline (Institute for Vision Research, Iowa City, IA). MEE cases and Australian cohorts were analyzed with exome sequencing as previously described [12, 31]. The MEE control data was extracted from a deidentified exome repository derived from individuals who had an eye exam but did not have a diagnosis of glaucoma. The cohort of PDS patients from the NYEEI was tested for mutations in the *MRAP* gene using standard Sanger sequencing as previously described [32].

### Variant filtering and mutation analysis strategy

We excluded variants from our analysis of the Iowa cohorts if they had a variant quality of less than 50, or if fewer than 20% of the reads supported the variation. Such variations are a common source of false positives that do not validate upon Sanger sequencing. A total of 17,253 genes met criteria for whole exome analyses. We judged variants with a minor allele frequency > 2.5% in gnomAD populations (Non-Finnish European, African, and South Asian) or present in > 2.5% of our control patients to be too common to cause PDS and these variants were excluded from the analysis.

The Iowa cohort of 201 PDS patients and 362 controls in the genetic study was further filtered based on their exome sequences. Two subjects (1 PDS patient and 1 control) were excluded from analysis due to relatedness with another study subject. We also conducted a principal components analysis of the remaining of 200 PDS patients and 361 controls that had complete clinical records and exome sequence available as previously described [33]. We identified two PDS patients and 2 control patients as outliers for the first two principal components (Supplemental Figure 1). Three of the outliers were of African ancestry (two PDS patients and one control patient), while one outlier was a non-Hispanic white control patient. These four outliers were excluded from our study and our subsequent genetic studies were conducted on the exomes from the remaining 198 PDS patients and 359 controls from Iowa.

### Mutation analysis and statistics

We conducted one primary analysis and three secondary analyses on the mutation data available from our cohorts of PDS patients (*n* = 198) and control subjects (*n* = 359) from Iowa.

#### Primary analysis

The frequency of *loss-of-function* mutations in each of 5 candidate genes was compared between the PDS and control groups using Fisher’s exact test. Loss-of-function mutations were defined as premature termination mutations, frameshift mutations, and canonical splice site mutations. The five top candidate genes for causing human PDS were genes that have been previously shown to cause pigment dispersion in mice (*TYRP1* [10], *GPNMB* [10], *LYST* [21], *DCT* [19], and *MITF* [19]). The frequency of loss-of-function mutations in each gene was analyzed separately and a Bonferroni corrected *p*-value of 0.05 / 5 genes examined = 0.01 was used as a threshold for significance.

#### Secondary analysis

The frequency of *loss-of-function* mutations in two additional sets of genes were compared between the PDS and control groups in additional, secondary analyses. As secondary analyses, these investigations were conducted for hypothesis generation rather than establishing statistical significance. Consequently, we did not employ multiple measures corrections for these analyses.

#### Secondary analysis− 1

We identified *loss*-*of*-*function* mutations in a set of 21 additional genes (Supplemental Table 1)*.* We compared the frequency of these mutations in the case cohort with the frequency in the control cohort. The frequency of loss-of-function mutations in each of the 21 genes were analyzed separately and uncorrected *p*-values were calculated using Fisher’s exact test. Uncorrected p-values were calculated to prioritize candidate genes identified with this hypothesis generation set of experiments.

#### Secondary analysis-2

We identified all non-silent mutations in all of the genes in the exome that passed quality control filtering (*n* = 17,253). Non-silent mutations include: missense, nonsense, frameshift, canonical spice site, and in-frame deletion/insertion mutations. After manual inspection of in-frame deletions and insertions, we determined that variants of this nature with less than 35% of the total overlapping reads should be removed from the analysis due to a high number of false positive calls with less frequently observed variations. Loss-of-function mutations with a minor allele frequency reported to be greater than 2.5% in gnomAD [34] were removed. We calculated the non-silent mutation burden in each of the 17,253 genes for the PDS cohort using the SKAT-O software package and the SKATBinary algorithm [35].

### Immunohistochemical analysis of human eyes

Human donor eyes were obtained from the Iowa Lions Eye Bank (Iowa City, IA). Consent for research was obtained from the donor’s next of kin in all cases, and all experiments were performed in accordance with the Declaration of Helsinki. Eyes were fixed for two hours in 4% paraformaldehyde in 10 mM PBS (pH 7.4) within 6–8 h after death. Anterior segments from all eyes were cryoprotected by passing through a sucrose gradient before being embedded in 20% sucrose in Optimal Cutting Temperature compound (Ted Pella, Redding, CA) [36]. Sections of 7 μm thickness were collected from each sample using a Microm H505E cryostat (Waldorf, Germany) and mounted on Superfrost plus slides (Ted Pella, Redding, CA). Immunofluorescence procedures were performed as previously described [37]. Sections were blocked for 15 min using a PBS solution with 1 mg/mL bovine serum albumin. Sections were then incubated in the primary antibody solution for 1 h, followed by rinsing three times with PBS and incubation in the appropriate Alexa 488– and Alexa 546-conjugated secondary antibodies (Invitrogen, Eugene, OR) for 30 min. Sections were counterstained with 4′,6-diamidino-2-phenylindole (DAPI), washed three times for five minutes in PBS, and coverslipped with Aquamount. The polyclonal rabbit anti-human MRAP antibody (OAAB06581, Aviva Systems Biology, San Diego, CA) was used at a concentration of 9μg/mL and a mouse anti-human collagen IV monoclonal antibody (M3F7, Development Studies Hybridoma Bank, University of Iowa, Iowa City, IA) was used at a concentration of 1μg/mL. Sections were photographed using an Olympus BX41 fluorescence microscope with a SPOT RT camera.

### Statistical analyses

Power calculations were conducted to evaluate our ability to detect an enrichment of loss-of-function mutations in PDS patients when compared with control subjects in five candidate genes (*TYRP1*, *GPNMB*, *LYST*, *DCT*, and *MITF*). With the cohort in our study (198 PDS patients and 359 controls), we had 80% power to detect a statistically significant skew in loss-of-function mutations at significance level 1% if they occur at a frequency of 3.2% or greater in the PDS population and 0% in the control population. Fisher’s exact test was used with a threshold of *p* < 0.05 / 5 (*p* < 0.01) for significance in the primary analysis. Secondary analyses were not corrected for multiple measures as they were hypothesis generating experiments. *MRAP* mutations were analyzed in a second cohort of 88 PDS patients from NYEEI; 150 PDS patients and 1500 controls from MEEI; and 177 PDS patients and 145 controls from Australia. This cohort had greater than 80% power to detect a statistically significant increase in the frequency of non-synonymous mutations at significance level 5% if they occurred at a frequency of 3% or greater in the PDS population and 0% in the control population. The combined data from these cohorts were analyzed using Cochran-Mantel-Haenszel test with continuity correction [38, 39].

## Supplementary Information

**Additional file 1: Supplementary Table 1.** Secondary analysis of candidate genes. The symbols, names, and functions of genes included in the secondary analysis due to their role in melanin synthesis or melanosome structure.

**Additional file 2: Supplementary Table 2.** Secondary analysis: mutations detected in candidate genes. Single instances of loss-of-function mutations were detected in three of the candidate genes in the secondary analysis.

**Additional file 3: Supplementary Table 3.** Secondary analysis: mutations detected in whole exome analysis.

**Additional file 4: Supplementary Figure 1.** Principal components analysis of the PDS and control cohort from Iowa. The entire Iowa cohort of was investigated with principal components analysis of SNPs identified from exome sequences. The distribution of the first and second principal components (PC1 and PC2) are shown where the 200 PDS patients are each represented with red dots and the 361 controls are each represented with blue dots. PANEL A. Four individuals that are outliers for principal components 1 and 2 are shown with the green arrows. A tight cluster of the remaining 557 study subjects are located within a small red box. PANEL B. An exploded view of the red box in panel A showing the distribution of 557 members of the Iowa cohort with the 4 outliers removed.

## Data Availability

The datasets used and/or analyzed during the current studdy are available at NCBI dbGaP with accession numbers XXXX.

## Abbreviations

PDS: Pigment dispersion syndrome
IA: Iowa
IOP: Intraocular pressure
MEE: Massachusetts Eye and Ear
NYEEI: New York Eye and Ear Infirmary
OHT: Ocular hypertension
PCA: Principal components analysis
PG: Pigmentary glaucoma
PMEL: Premelanosome protein gene
TYRP1: Tyrosinase-related protein 1 gene
GPNMB: Glycoprotein nmb gene
LYST: Lysosome trafficking regulator gene
DCT: Dopachrome tautomerase gene
MITF: Melanocyte inducing transcription factor gene
MC1R: Melanocortin 1 receptor gene
TYR: Tyrosinase gene
SLC45A2: Solute carrier family 45 member 2 gene
MRAP: Melanocortin 2 receptor accessory protein gene
NYEEI: New York Eye and Ear Infirmary
MEE: Massachusetts Eye and Ear
