# Supplementary material for: Exome-based investigation of the genetic basis of human pigmentary glaucoma
Source: BMC Genomics. 2021 Jun 26;22:477. doi: 10.1186/s12864-021-07782-0 (PMC8235805; doi:10.1186/s12864-021-07782-0)
Supplement: Supplementary file 1 — Additional file 1: Supplementary Table 1. Secondary analysis of candidate genes. The symbols, names, and functions of genes included in the secondary analysis due to their role in melanin synthesis or melanosome structure. [file 12864_2021_7782_MOESM1_ESM.docx]

| **SUPPLEMENTARY TABLE 1** | | |
| --- | --- | --- |
| **Gene Symbol** | **Gene Name** | **Gene Function** |
| *AGRP* | Agouti related neuropeptide | Antagonist to melanocortin receptors (MC3R and MC4R). |
| *ATP6V1A* | ATPase H+ transporting V1 subunit A | A component of the enzyme that promotes acidification of intracellular organelles. |
| *ATP6V1B2* | ATPase H+ transporting V1 subunit B2 | A component of the enzyme that promotes acidification of intracellular organelles. |
| *COMT* | Catechol-O-methyltransferase | Degradation of metabolites produced during melanin synthesis. |
| *CYB5R3* | Cytochrome b5 reductase 3 | Encodes an enzyme that helps to detoxify intermediates produced during melanin synthesis |
| *EN2* | Engrailed homeobox 2 | Encodes a homeobox gene and is located within a region of chromosome 7q that was previously linked with human pigment dispersion syndrome (GPDS) |
| *GPR143* | G protein-coupled receptor 143 | Mutations in GPR143 (OA1) cause ocular albinism (Nettleship-Falls ocular albinism). |
| *LAMP1* | Lysosomal associated membrane protein 1 | Encodes membrane proteins of lysosomes which have similarities with melanosomes |
| *LAMP2* | Lysosomal associated membrane protein 2 | Encodes membrane proteins of lysosomes which have similarities with melanosomes |
| *MC1R* | Melanocortin receptor 1 | Encodes a receptor for melanocyte stimulating hormone, which promotes production of eumelanin. Mutations promote pheomelanin production and lighter pigmentation. |
| *MIF* | Macrophage migration inhibitory factor | Encodes an enzyme that helps to detoxify intermediates produced during melanin synthesis |
| *MLANA* | Melan-A | Encodes a protein that promotes trafficking and processing of PMEL which is critical in melanosome formation |
| *MNX1* | Motor neuron and pancreas homeobox 1 | Encodes a homeobox gene and is located within a region of chromosome 7q that was previously linked with human pigment dispersion syndrome (GPDS) |
| *PAXIP1* | PAX interacting protein 1 | Encodes a protein that regulates PAX2, a homeobox gene, and is located within a region of chromosome 7q that was previously linked with human pigment dispersion syndrome (GPDS) |
| *PMEL* | Premelanosome protein | Encodes a melanocyte-specific, glycoprotein that has a role in developing melanosomes |
| *POMC* | Proopiomelanocortin | Encodes a pre-protein that may be cleaved to form melanocortin and stimulate melanocytes |
| *RACK1* | Receptor for activated C kinase 1 |  |
| *SHH* | Sonic Hedgehog | Encodes a homeobox gene and is located within a region of chromosome 7q that was previously linked with human pigment dispersion syndrome (GPDS) |
| *SLC45A2* | Solute carrier family 45 member 2 | Encodes a transporter protein that mediates melanin synthesis and mutations are associated with oculocutaneous albinism type 4 (OCA4) |
| *TYR* | Tyrosinase | Encodes a protein that has enzymatic function in melanin synthesis. Mutations are associated with oculocutaneous albinism type 1A and 1B (OCA1). |
| *VAT1* | Vesicle amine transport 1 | Encodes a protein that is involved in vesicle transport |
|  |  |  |
| **Supplementary Table 1. Secondary analysis of candidate genes.** The symbols, names, and functions of genes included in the secondary analysis due to their role in melanin synthesis or melanosome structure. | | |
|  |  |  |
