# Supplementary material for: Exome-based investigation of the genetic basis of human pigmentary glaucoma
Source: BMC Genomics. 2021 Jun 26;22:477. doi: 10.1186/s12864-021-07782-0 (PMC8235805; doi:10.1186/s12864-021-07782-0)
Supplement: Supplementary file 2 — Additional file 2: Supplementary Table 2. Secondary analysis: mutations detected in candidate genes. Single instances of loss-of-function mutations were detected in three of the candidate genes in the secondary analysis. [file 12864_2021_7782_MOESM2_ESM.docx]

| **SUPPLEMENTARY TABLE 2** | | | | | | | | | |
| --- | --- | --- | --- | --- | --- | --- | --- | --- | --- |
|  |  |  |  |  | |  | |  |  |
|  |  |  |  |  | |  | |  |  |
|  |  |  |  | **Iowa PDS Cohort** | | **Iowa Normal Cohort** | |  | **gnomAD** |
|  | **Loss of function mutation** | | | **n = 198** | | **n = 359** | |  | **European (non-Finnish)** |
| **Gene** | **SNP ID** | **Mutation** | **Encoded Protein** | **Instances** | **Genotype frequency** | **Instances** | **Genotype frequency** | **P-value** | **Genotype frequency** |
| *MC1R* | rs555179612 | NM_002386.3:c.537dupC | p.Ile180HisfsTer59 | 1 | 0.51% | 0 | 0.00% | 0.36 | 0.10% |
| *SLC45A2* | rs775387808 | NM_001012509.4:c.264delC | p.Gly89AspfsTer24 | 0 | 0.00% | 1 | 0.28% | >0.99 | 0.01% |
| *TYR* | rs61753256 | NM_000372.5:c.346C>T | p.Arg116Ter | 1 | 0.51% | 0 | 0.00% | 0.36 | 0.001% |
|  | | | | | | | | | |
|  | | | | | | | | | |
| **Supplementary Table 2: Secondary analysis: mutations detected in candidate genes.** Single instances of loss-of-function mutations were detected in three of the candidate genes in the secondary analysis. | | | | | | | | | |
