# Supplementary material for: Exome-based investigation of the genetic basis of human pigmentary glaucoma
Source: BMC Genomics. 2021 Jun 26;22:477. doi: 10.1186/s12864-021-07782-0 (PMC8235805; doi:10.1186/s12864-021-07782-0)
Supplement: Supplementary file 3 — Additional file 3: Supplementary Table 3. Secondary analysis: mutations detected in whole exome analysis. [file 12864_2021_7782_MOESM3_ESM.docx]

| **SUPPLEMENTARY TABLE 3** | | | |
| --- | --- | --- | --- |
|  |  | |  |
|  |  | |  |
|  | **Non-silent mutations** | |  |
|  | **PDS cohort** | **Control cohort** | **Uncorrected**  **p-value** |
| **Gene** | **n=198** | **n=359** |  |
| *MRAP* | 4 (2.0%) | 0 | 0.016 |
| *TPO* | 17 (8.6%) | 7 (1.9%) | 0.00065 |
| *F10* | 11 (5.6%) | 4 (1.1%) | 0.0041 |
| *GET4* | 24 (12%) | 14 (3.9%) | 0.00036 |
| *KRT37* | 14 (7.1%) | 5 (1.4%) | 0.00086 |
| *ADCY5* | 18 (9.1%) | 15 (4.2%) | 0.024 |
|  |  |  |  |
|  |  |  |  |
| **Supplementary Table 3: Secondary analysis: mutations detected in whole exome analysis.** | | | |
