# Supplementary figures and images for: Exome-based investigation of the genetic basis of human pigmentary glaucoma
Source: BMC Genomics. 2021 Jun 26;22:477. doi: 10.1186/s12864-021-07782-0 (PMC8235805; doi:10.1186/s12864-021-07782-0)

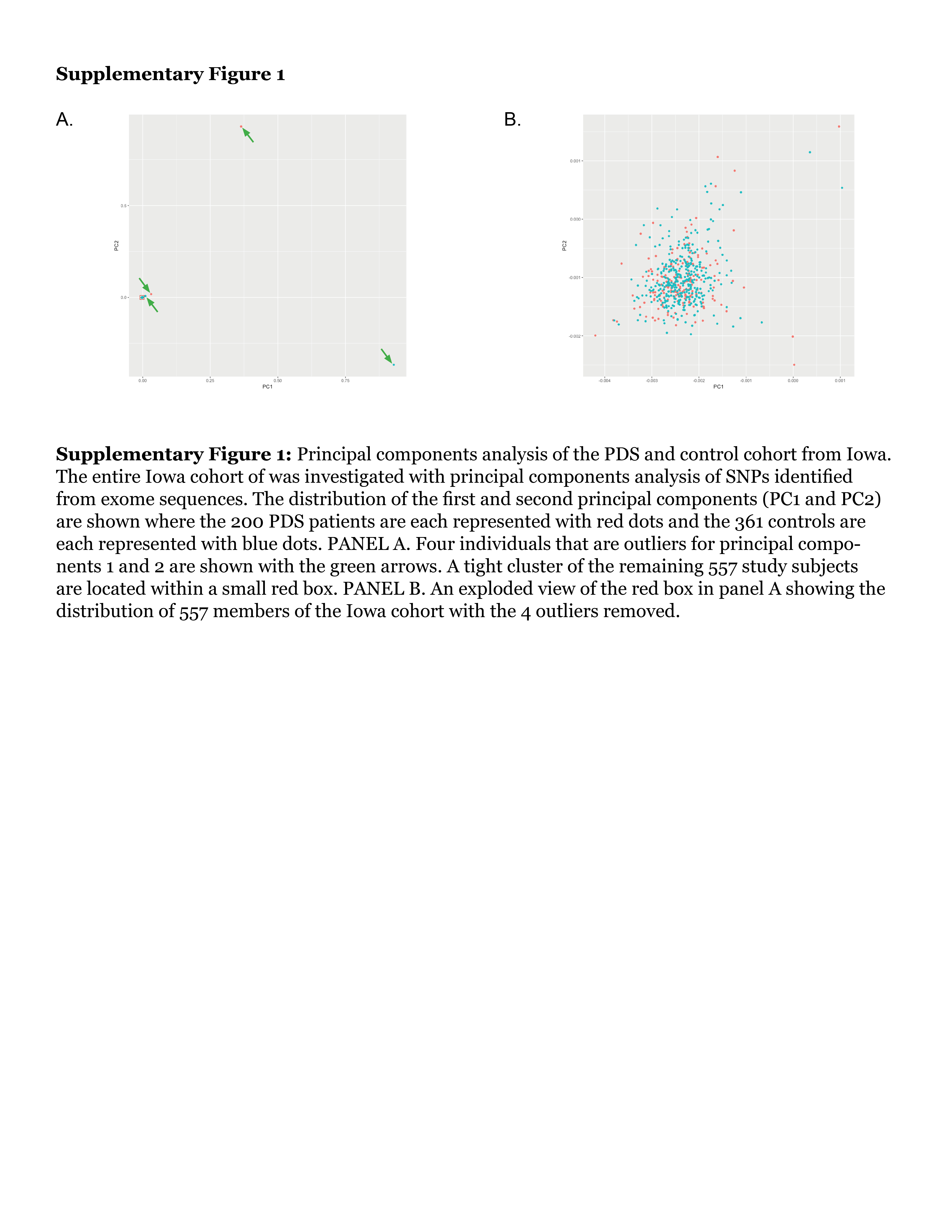

Supplement: Supplementary file 4 — Additional file 4: Supplementary Figure 1. Principal components analysis of the PDS and control cohort from Iowa. The entire Iowa cohort of was investigated with principal components analysis of SNPs identified from exome sequences. The distribution of the first and second principal components (PC1 and PC2) are shown where the 200 PDS patients are each represented with red dots and the 361 controls are each represented with blue dots. PANEL A. Four individuals that are outliers for principal components 1 and 2 are shown with the green arrows. A tight cluster of the remaining 557 study subjects are located within a small red box. PANEL B. An exploded view of the red box in panel A showing the distribution of 557 members of the Iowa cohort with the 4 outliers removed. [file 12864_2021_7782_MOESM4_ESM.jpg]
